# Vaccination Strategies and Research Gaps in Hepatitis E Virus for Special Populations

**DOI:** 10.3390/vaccines13060621

**Published:** 2025-06-09

**Authors:** Meng Wang, Binwei Duan, Mengcheng Liu, Yuxuan Zhang, Feng Wu, Guangming Li, Yabo Ouyang

**Affiliations:** 1 Department of General Surgery Center, Beijing YouAn Hospital, Capital Medical University, Beijing Institute of Hepatology, Beijing 100069, China; wangm@mail.ccmu.edu.cn (M.W.); bw_duan317@ccmu.edu.cn (B.D.); zhangyx_991020@mail.ccmu.edu.cn (Y.Z.); wufeng@mail.ccmu.edu.cn (F.W.); 2 Clinical Center for Liver Cancer, Capital Medical University, Beijing 100069, China

**Keywords:** Hepatitis E virus, vaccination strategies, special populations, HEV epidemiology, pregnant women, immunocompromised individuals, chronic liver disease, the elderly

## Abstract

Background: Hepatitis E virus (HEV) infection poses a significant health risk across diverse demographic groups, particularly among pregnant women, immunocompromised individuals, patients with chronic liver disease, and the elderly. The global epidemiology of HEV reveals distinct patterns of prevalence, transmission, and disease severity among these populations, necessitating targeted vaccination strategies. The licensing of the Hecolin (HEV 239) vaccine offers promise, but gaps in clinical trial data and varying immune responses in high-risk groups challenge its widespread applicability. Scope: This review synthesizes data on HEV’s epidemiology, discusses the susceptibility of vulnerable populations, evaluates the efficacy and safety of HEV 239, and highlights the urgent need for clinical research tailored to these groups. Key findings underscore the complexity of vaccine response influenced by immunological, physiological, and environmental factors. Additionally, potential advancements in vaccine technology, including the development of broad-spectrum vaccines and innovative delivery systems, are discussed as future directions. Strategies: Addressing regulatory, economic, and logistical barriers remains crucial for effective HEV vaccination programs. A multidisciplinary approach integrating public health policy, rigorous clinical evaluations, and collaborative frameworks is essential to ensure equitable access to HEV vaccination, ultimately improving health outcomes on a global scale.

## 1. Introduction

Hepatitis E virus (HEV), a significant global health issue, belongs to the Hepeviridae family.It primarily spreads through the fecal–oral route, especially in regions with poor sanitation, causing waterborne outbreaks [1]. Zoonotic and food-borne transmission are increasingly recognized, particularly in developed nations [2]. HEV exhibits significant genotypic variability, with at least four major genotypes (1–4) infecting humans [3,4]. These genotypes differ in their geographic distribution, transmission routes, and clinical manifestations [5]. Genotypes 1 and 2 are primarily found in developing countries and are transmitted through contaminated water, causing large outbreaks of acute hepatitis [3,4]. Genotypes 3 and 4 are more common in developed countries and are transmitted zoonotically, primarily through the consumption of undercooked pork or wild game [3,4].

HEV affects around 20 million people globally each year, with the highest incidence in developing regions such as Asia, Africa, and Latin America, where inadequate sanitation facilitates the virus’s spread [5]. Socioeconomic factors, including poverty and overcrowding, further elevate the infection risk [6]. HEV accounts for a substantial number of acute infections, hospitalizations, and deaths annually [5]. Pregnant women and individuals with liver diseases are at heightened risk [5,7]. The rise in affected populations and outbreaks in both endemic and non-endemic areas highlights the need for better control measures [1].

HEV symptoms range from asymptomatic to symptomatic infections, with jaundice being common [2]. However, clinical severity varies. Pregnant women are more likely to develop severe outcomes, such as fulminant hepatitis and complications, increasing maternal and fetal mortality [2,7]. Immunocompromised individuals, including organ transplant recipients, HIV patients, and those with autoimmune diseases, are at risk of chronic infection leading to liver fibrosis and cirrhosis [1,2,8,9]. Patients with pre-existing liver conditions are also prone to severe outcomes, including liver failure [8,9]. The elderly also face increased risks from HEV infection, often experiencing more severe symptoms and complications [10,11]. These risks emphasize the need for effective vaccine safety and efficacy in these groups [7].

Vaccination is key in controlling HEV transmission and reducing morbidity and mortality [6]. Hecolin (HEV 239) is the only licensed vaccine available in China [6,12]. Clinical trials have proven its efficacy and safety in the general population [13,14,15,16,17], and vaccination campaigns show promise in achieving herd immunity and supporting broader public health goals [7,18]. The WHO Position Paper on Hepatitis E provides critical recommendations on HEV vaccine use, particularly in regions with endemic outbreaks [19]. Despite its proven efficacy, HEV 239 has limitations, including the lack of widespread regulatory approval outside China and limited data on its effectiveness in special populations such as pregnant women, patients with chronic liver disease, and immunocompromised individuals [3,7,20].

Current vaccine data are insufficient for high-risk groups due to their exclusion from initial clinical trials and potentially altered immune responses [3,7,21]. There are significant gaps in safety and efficacy evidence for HEV vaccines in specific populations, including pregnant women, those with chronic liver disease (CLD), and the elderly [7,21]. A comprehensive assessment of the available data and the identification of research gaps is crucial to inform vaccination strategies for these vulnerable groups.

The WHO report on HEV vaccines, presented in March 2024, primarily focuses on general vaccine efficacy [22]. In contrast, this review emphasizes the challenges and opportunities associated with HEV vaccination in special populations, such as pregnant women and immunocompromised individuals, which have been underrepresented in global vaccine discussions. By synthesizing existing epidemiological data, understanding immunological mechanisms, and analyzing clinical outcomes, the review aims to provide a comprehensive overview of HEV vaccination in special populations. The objective is to guide future research and inform the development of tailored vaccination strategies to improve public health outcomes globally [7,23].

The review was conducted by searching relevant databases including PubMed and Web of Science, with a focus on studies published from 2005 onward. The review was not registered with PROSPERO because it is not a systematic review but rather an overview aimed at synthesizing existing knowledge.

## 2. Pregnant Women

### 2.1. Immunological Changes During Pregnancy and Associated HEV Risk

#### 2.1.1. Immune Modulation (Th2 Bias, Cytokine Shifts) and Its Impact on HEV Replication

Pregnancy induces significant immunological changes, including a shift towards a T helper 2 (Th2) immune response, expansion of regulatory T cells, and altered cytokine profiles [24]. This immune modulation is crucial for maintaining fetal tolerance but can also affect susceptibility to certain infections, including HEV [2]. The Th2 bias, characterized by the increased production of cytokines such as IL-4, IL-5, IL-10, and IL-13, is thought to suppress the Th1 response, which is essential for clearing intracellular pathogens like HEV [25,26]. This shift can impair viral clearance, leading to prolonged HEV infection and increased viremia in pregnant women [2]. Clinical data indicate a correlation between these immune shifts and higher HEV viral loads in pregnant women [2].

#### 2.1.2. Mechanistic Links to Severe Outcomes (Fulminant Hepatitis, Obstetric Complications)

HEV infection during pregnancy is associated with severe outcomes, including fulminant hepatitis, placental dysfunction, preterm birth, maternal mortality, and obstetric complications [23,24,25,26]. The pathophysiological mechanisms linking HEV infection to these severe outcomes involve a complex interplay of viral load, immune evasion, and inflammatory responses [2,27]. High viral loads can exacerbate liver damage, leading to fulminant hepatitis [2,28]. The altered immune response during pregnancy may impair the ability to control HEV replication, resulting in increased viral titers and more severe liver injury [2,29]. Moreover, inflammatory responses, driven by the host’s immune system, can contribute to placental dysfunction and obstetric complications such as preterm birth and maternal mortality [2,30]. Epidemiological studies have consistently shown higher mortality rates in pregnant women infected with HEV, particularly in endemic regions [7,31,32,33]. In developing countries, HEV genotypes 1 and 2 cause large outbreaks and affect young subjects, resulting in significant mortality in pregnant women [34,35]. Specifically, acute hepatitis, most of it likely hepatitis E, is responsible for ≈9.8% of pregnancy-associated deaths [31]. If these numbers are representative of southern Asia, as many as 10,500 maternal deaths each year in this region alone may be attributable to hepatitis E and could be prevented by using existing vaccines [31].

### 2.2. Preclinical Insights and Animal Model Data

#### 2.2.1. Liposome-Encapsulated Antigen Studies and Antibody Responses

Preclinical studies have evaluated liposome-based HEV vaccines in animal models, such as mice and rabbits, to assess their immunogenicity and safety [36,37]. Liposomes, which are spherical vesicles composed of lipid bilayers, can encapsulate and deliver antigens to immune cells, potentially enhancing the immune response [36,38]. Studies have compared the immunogenicity of conventional HEV vaccines with novel liposome-based formulations, focusing on antibody titers and T-cell responses [36,38]. The results suggest that liposomal delivery may offer advantages, including enhanced stability and prolonged antigen presentation, leading to improved immune responses [36,39]. For example, a study in pregnant mice demonstrated that a single dose of recombinant Neutralizing Epitope protein (rNEp) encapsulated in liposomes was safe and highly immunogenic, resulting in significantly higher anti-HEV titers compared to non-pregnant mice [36]. The higher antibody response in pregnant mice was independent of the genetic makeup of the host but immunogen-driven [36,39].

#### 2.2.2. Maternal–Fetal Antibody Transfer and Accelerated Immunization Schedules

An animal study has investigated maternal–fetal antibody transfer following HEV vaccination to assess the potential for protecting newborns [37]. Maternal antibodies, particularly IgG, can cross the placenta and provide passive immunity to the fetus [37,40]. Studies in pregnant rabbits have shown that vaccination with HEV 239 vaccine can induce high titers of anti-HEV protective antibodies in a short period of time, which protect the pregnant rabbits from HEV infection and adverse pregnancy outcomes [37]. In addition, the immunized rabbits transfer maternal antibodies to pups through the placenta and breast milk, which protect neonates against HEV infection [37]. Accelerated vaccination schedules, involving shorter intervals between doses, have also been evaluated to determine if they can improve neonatal protection [37]. These findings have implications for designing vaccination strategies in pregnant women, as they suggest that maternal vaccination can provide protection to both the mother and the newborn [37].

### 2.3. Clinical Data Gaps and Ethical Considerations

#### 2.3.1. Limitations of Current Clinical Trial Data in Pregnant Populations

Current clinical trial data on HEV vaccines in pregnant populations are limited, leading to significant gaps in our understanding of vaccine safety and efficacy in this vulnerable group [7,33]. Many initial HEV vaccine studies excluded pregnant women due to ethical and regulatory restrictions [7,33,41]. The scarcity of Phase III trial data in pregnant women highlights the need for more comprehensive research. [7,33]. Challenges in assessing vaccine safety and efficacy arise from the ethical dilemmas associated with conducting clinical trials during pregnancy, including concerns about potential risks to the fetus and the need for informed consent [7,33,42]. Post-marketing surveillance in vaccinated pregnant women is crucial for monitoring long-term safety and efficacy outcomes [7,33,43].

#### 2.3.2. Ethical Challenges and Considerations for Vaccine Trials During Pregnancy

Vaccine trials during pregnancy present complex ethical dilemmas, requiring careful consideration of the risk–benefit assessment, informed consent, and fetal safety concerns [33,44]. Regulatory frameworks, such as those established by the FDA and EMA, provide guidelines for conducting vaccine trials in pregnant women, but ethical challenges remain [33,45]. Strategies to ensure ethical trial conduct while advancing research include thorough preclinical testing, comprehensive informed consent processes, and strict monitoring of maternal and fetal outcomes [33,46]. Comparing regulatory frameworks (e.g., FDA, EMA) for vaccine trials in pregnant women, and proposing strategies to ensure ethical trial conduct while advancing research are essential [33,47].

### 2.4. Proposed Strategies for Vaccination

#### 2.4.1. Vaccination Prior to Conception Versus Early Pregnancy Administration

The optimal timing of HEV vaccination in women of childbearing age is a critical consideration, with two main strategies: vaccination prior to conception versus early pregnancy administration [33]. Vaccination prior to conception offers the advantage of providing protection before potential exposure to HEV during pregnancy, reducing the risk of severe outcomes [33]. However, this strategy requires identifying high-risk women before they become pregnant, which can be logistically challenging [33]. Early pregnancy administration may be considered in situations where pre-conception vaccination is not feasible, but it raises concerns about potential risks to the developing fetus, particularly during the first trimester [33,41]. Evidence from other vaccines, such as influenza and Tdap, can inform HEV vaccination timing decisions, as these vaccines have been shown to be safe and effective during pregnancy [33,48]. Weighing the benefits and risks of pre-pregnancy vaccination vs. early pregnancy administration is essential for developing appropriate vaccination guidelines [33].

#### 2.4.2. Design of Multi-Center, Randomized Controlled Trials with Strict Monitoring

Future clinical trials evaluating HEV vaccines in pregnant women should incorporate key elements such as clear inclusion criteria, well-defined safety endpoints, and comprehensive immunogenicity assessments [33,41]. Multi-center collaborations are essential to ensure diverse population representation, enhancing the generalizability of trial results [33,41]. Strict monitoring protocols should be implemented to track maternal–fetal outcomes and assess long-term neonatal immunity [33,41]. Proposed strategies include designing multi-center trials for high-risk groups, optimizing timing strategies (e.g., vaccination prior to exposure), and emphasizing the need for multi-center collaborations to ensure diverse population representation [33,41].

## 3. Immunocompromised Patients

### 3.1. Clinical Significance of HEV Infection in Immunocompromised Hosts

#### 3.1.1. Increased Risk of Chronicity and Rapid Progression to Liver Cirrhosis

Hepatitis E virus (HEV) infection in immunocompromised individuals poses a far greater clinical challenge than in immunocompetent hosts [49]. In otherwise healthy individuals, HEV infection is typically self-limiting; however, in patients with impaired immune function, the virus can persist, leading to chronic infection [49,50]. This persistence is largely a consequence of an inability to clear the virus effectively due to deficient cellular and humoral immune responses [51]. In such settings, ongoing viral replication perpetuates low-grade inflammation that eventually results in progressive liver fibrosis and rapid progression to cirrhosis [52,53]. Studies have shown that patients who develop chronic HEV infection—particularly those on long-term immunosuppressive regimens—are at a significantly increased risk of accelerated liver decompensation and cirrhotic transformation [7,54].

The biological basis underlying this phenomenon is multifactorial. Persistent infection leads to continuous activation of hepatic stellate cells and subsequent deposition of extracellular matrix proteins [55,56]. This cascade of events contributes to irreversible architectural disruption of liver tissue [49]. Moreover, the ability of HEV to persist in the presence of immunosuppression appears to correlate with the degree of immune inhibition; patients with more profound immune suppression tend to experience more rapid progression of liver fibrosis [55,56]. Clinical studies among liver transplant recipients have documented a higher incidence of persistent HEV RNA detection and a strikingly accelerated course toward cirrhosis compared to immunocompetent individuals [49,57]. Such findings underscore the critical need for targeted strategies to prevent or mitigate chronic HEV infections in this vulnerable patient group [55,56].

#### 3.1.2. Specific Risks in Organ Transplant Recipients and HIV-Infected Patients

Among the immunocompromised, organ transplant recipients comprise a high-risk subgroup for chronic HEV infection [58,59]. In these patients, the routine administration of immunosuppressive drugs—essential for preventing allograft rejection—creates a milieu in which the virus can evade immune surveillance [58]. A large retrospective study on liver transplant recipients revealed a notable cumulative incidence of de novo HEV infection post-transplantation, which was further associated with baseline indicators such as liver failure and biochemical abnormalities [57]. These patients not only face the risk of developing chronic HEV infection but also exhibit a rapid progression to cirrhosis, which substantially complicates post-transplant management and adversely affects long-term graft survival [60,61].

HIV-infected patients represent another subgroup at elevated risk. Even in the era of modern antiretroviral therapy, where partial immune reconstitution is achievable, many HIV-infected individuals continue to experience immune deficits that hinder the clearance of HEV [62,63]. Chronic HEV infection in these patients has been documented despite improvements in CD4+ T cell counts, suggesting that numerical recovery does not necessarily equate to full functional immune competence [62,64]. Persistent viral replication in this setting may lead to chronic hepatic inflammation and fibrosis, further exacerbating the health burden in individuals already contending with HIV-related complications [2,65]. Consequently, both organ transplant recipients and HIV-infected patients require special attention when it comes to the prevention and management of HEV infection due to their distinct underlying immunological impairments and the heightened risk of sustained liver injury [8,23].

### 3.2. Impact of Immunosuppressive Therapies on Vaccine Response

#### 3.2.1. Effects of Drugs (e.g., Tacrolimus, MMF) on Immunogenicity

The efficacy of vaccination strategies in immunocompromised patients is significantly affected by the concurrent use of immunosuppressive medications [66,67]. Drugs such as tacrolimus and mycophenolate mofetil (MMF) are widely employed in post-transplant regimens and other conditions requiring immunosuppression [68,69]. Tacrolimus, a calcineurin inhibitor, exerts its effects by blocking the production of interleukin-2 and other cytokines essential for T cell activation, thereby inhibiting the proliferation and function of T lymphocytes [69,70]. Similarly, MMF targets the de novo synthesis of guanine nucleotides by inhibiting inosine monophosphate dehydrogenase, a critical enzyme needed for the proliferation of both T and B lymphocytes [71,72]. The net result is a dampened immune response that compromises the generation of robust antibody titers following vaccination [3,54].

These pharmacological effects translate into suboptimal vaccine-induced immunogenicity [67,73]. Studies assessing vaccine responses in recipients of immunosuppressive therapy have consistently demonstrated lowered seroconversion rates, reduced antibody titers, and impaired cellular immunity [73]. In the context of HEV vaccination, these limitations may lead to insufficient protection against infection despite vaccine administration [67,68]. The inhibitory actions of these agents not only hinder the initial immune priming but also negatively affect the generation of memory cells, which are essential for long-term protection [66,67]. As a result, immunocompromised patients are at risk of both reduced immediate vaccine efficacy and a more rapid decline in protective antibody levels over time [72,73].

#### 3.2.2. Mechanisms Underlying Impaired Antibody and T Cell Responses

At the cellular level, the impaired vaccine response in immunosuppressed individuals is multifaceted [74]. One central mechanism is the disruption of antigen presentation. Immunosuppressive drugs can diminish the number and functionality of antigen-presenting cells (APCs), including dendritic cells and macrophages, thereby limiting the effective presentation of the vaccine antigen to T lymphocytes [75,76]. With suboptimal antigen presentation, the activation and differentiation of CD4+ helper T cells are adversely affected [77]. These helper T cells play a pivotal role in orchestrating a coordinated immune response by aiding both the B cell-mediated humoral response and the activation of cytotoxic T cells [77].

Furthermore, immunosuppressive therapy directly impairs the clonal expansion of lymphocytes through the inhibition of key signaling pathways [74]. The blockade of T cell receptor (TCR) signaling prevents the full activation and proliferation of T cells, leading to fewer effector cells and a diminished pool of memory T cells [78]. The B cell compartment is similarly impacted. The inhibition of B cell proliferation and differentiation results in the decreased production of high-affinity, neutralizing antibodies [79]. In essence, the underlying mechanisms involve both a quantitative reduction in immune cell populations and a qualitative impairment of their functional capacity [75,76]. These combined effects culminate in a markedly reduced ability to mount both an immediate and a sustained immune response to vaccination, thereby challenging the overall efficacy of HEV immunization strategies in patients receiving immunosuppressants [2,80].

### 3.3. Strategies for Optimizing Immunization-Sequential “Vaccine–Immunomodulator” Approaches and Regimen Adjustments

Recognizing the attenuated vaccine responses observed in immunocompromised patients, researchers and clinicians are increasingly exploring innovative strategies to optimize immunization outcomes. One promising strategy is the implementation of sequential “vaccine–immunomodulator” approaches, which involve the judicious use of immunomodulatory agents in close coordination with vaccine administration [81,82]. These approaches are designed to temporarily enhance the host immune response at the time of vaccination by modulating the activity of the immune system without compromising the overall immunosuppressive regimen required for managing the patient’s underlying condition.

For example, in some clinical settings, a temporary reduction in or strategic adjustment of the dose of immunosuppressive medications around the time of vaccination has been proposed as a means to facilitate a more robust immunologic response [83]. Such adjustments must be carefully timed to avoid an unacceptable risk of graft rejection or disease flare while still permitting an adequate immune response to the vaccine antigen [84]. In parallel, the introduction of immunostimulatory adjuvants—such as MF59 or AS04—has been shown in other vaccine contexts to enhance antigen presentation and overall immunogenicity [85]. When applied to the HEV vaccine, these adjuvants may help overcome the inhibitory effects of immunosuppressive drugs by fostering a more favorable microenvironment for immune activation [3,7].

This sequential approach can be customized based on individual patient profiles. For instance, patients receiving high-dose calcineurin inhibitors may benefit from an immunization schedule that incorporates additional booster doses administered during periods of relatively lower immunosuppression [86]. In this way, the timing of vaccine administration is aligned with transient windows of opportunity when the patient’s immune response is most capable of responding adequately [87]. Although such protocols require rigorous clinical evaluation to balance efficacy with safety, they represent a promising avenue for enhancing the protective benefits of HEV vaccination in immunocompromised populations.

### 3.4. Future Clinical Trial Considerations

#### 3.4.1. Tailored Vaccine Schedules and Booster Strategies

Emerging evidence suggests that conventional vaccine schedules, as established in immunocompetent populations, are insufficient to elicit a durable and protective immune response in immunocompromised patients [88]. Future clinical trials must therefore adopt adaptive designs that evaluate tailored vaccine schedules for these high-risk groups. This may include the use of modified dosing regimens, such as administering double or even triple doses of the HEV vaccine, as well as incorporating additional booster shots at predetermined intervals.

In many immunocompromised patients, the initial vaccine response is suboptimal due to the underlying immune suppression; hence, booster strategies are critical to ensure that protective antibody levels are attained and maintained over time [89]. For instance, studies have suggested that booster immunizations can significantly enhance seroconversion rates and prolong the duration of protective immunity among transplant recipients and other immunosuppressed populations [90,91,92]. Furthermore, adaptive trial designs that allow for close monitoring and timely adjustment of booster schedules can refine our understanding of the optimal intervals between vaccine doses [93]. These tailored strategies will be instrumental in reducing the incidence of breakthrough infections and in mitigating the risk of progression to chronic liver disease in these patient cohorts [7,54].

#### 3.4.2. Incorporation of Individualized Immunosuppressive Adjustments in Trial Designs

In addition to revising vaccine schedules, future clinical trial designs must incorporate individualized adjustments in immunosuppressive therapy. Given that the degree and nature of immunosuppression vary widely among patients—depending on factors such as the type of organ transplant, the underlying disease severity, and the specific immunosuppressive drugs administered—it is imperative that clinical trials stratify participants based on these variables [7].

Personalized trial protocols could involve the real-time monitoring of immunologic biomarkers and pharmacodynamic assessments of immunosuppressant drug levels. For example, patients receiving high doses of tacrolimus may require different vaccination kinetics compared to those on lower doses or alternative agents like MMF [94]. Adjustments in immunosuppressive regimens, even if transient and controlled, could provide essential windows during which vaccine responsiveness is optimized [95]. Such individualized modifications should be integrated into clinical trial designs, with predefined criteria for reducing or temporarily altering immunosuppressive therapy in conjunction with vaccine administration [96]. This approach aims not only to boost the immunogenicity of the HEV vaccine but also to ensure that any alteration in immunosuppressive strategy does not compromise the control of the underlying condition [97].

Moreover, incorporating individualized immunosuppressive adjustments into trial protocols can facilitate the identification of potential biomarkers predictive of vaccine responsiveness [7]. A deeper understanding of the interplay between specific immunosuppressive agents and vaccine-induced immune responses will allow clinicians to design risk-adapted vaccination strategies that maximize benefit while minimizing the risk of adverse events such as graft rejection or opportunistic infections. Future trials that embed this level of customization are likely to yield clinically relevant data that can inform standardized practice guidelines and ultimately improve outcomes for immunocompromised patients [2,80].

## 4. Patients with Chronic Liver Disease

### 4.1. Risk of HEV Superinfection in Chronic Liver Disease

CLD patients are particularly susceptible to severe outcomes following HEV superinfection [7,98]. This heightened risk stems from a combination of compromised hepatic function and immune dysregulation inherent in CLD [11]. The presence of pre-existing liver damage impairs the liver’s ability to effectively clear the virus, while immune dysfunction can lead to an inadequate or dysregulated immune response, further exacerbating liver injury [99].

#### 4.1.1. Mechanisms of Accelerated Liver Decompensation and Failure

HEV superinfection in CLD patients can trigger a cascade of events that accelerate liver decompensation and failure [100]. The mechanisms involved include inflammation-mediated hepatocyte injury, mitochondrial dysfunction, and apoptosis [80].

HEV infection exacerbates pre-existing liver damage through several molecular pathways. The virus induces an inflammatory response, leading to the release of cytokines and recruitment of immune cells to the liver [2]. This heightened inflammation contributes to hepatocyte injury, further compromising liver function [101]. Additionally, HEV infection can disrupt mitochondrial function within liver cells, impairing energy production and promoting oxidative stress [80]. This mitochondrial dysfunction contributes to hepatocyte damage and apoptosis [80]. The cumulative effect of these processes is accelerated fibrosis progression and an increased likelihood of acute-on-chronic liver failure (ACLF) [100]. Studies have shown that HEV superinfection in patients with CHB-related cirrhosis is a risk factor for decompensated cirrhosis and an independent predictor of mortality [102,103,104].

#### 4.1.2. Importance of Patient Stratification (e.g., Child–Pugh Scores)

The effective management of HEV risk in CLD patients necessitates careful patient stratification based on clinical scoring systems such as the Child–Pugh score or Model for End-Stage Liver Disease (MELD) [4]. These tools help identify high-risk individuals who may benefit most from vaccination [98].

The Child–Pugh score assesses the severity of liver disease based on clinical parameters like ascites, encephalopathy, and bilirubin levels, while the MELD score incorporates creatinine and INR values for a more quantitative assessment [4]. Higher scores indicate more severe liver dysfunction and a greater risk of adverse outcomes following HEV superinfection [4].

Stratification allows clinicians to tailor their approach to HEV prevention and treatment. For instance, patients with Child–Pugh class A cirrhosis may be considered for HEV vaccination, while those with class C cirrhosis may require closer monitoring and alternative strategies [4]. In patients with CHB-related cirrhosis, HEV superinfection can lead to decompensated cirrhosis and increased mortality [100]. Stratification can improve outcome prediction and guide personalized therapeutic decisions, ensuring that resources are allocated effectively to those at highest risk [98].

### 4.2. Evaluation of Vaccine Safety and Initial Immunogenicity Data

Evaluating vaccine safety among CLD patients is crucial due to the potential for adverse effects, especially in advanced stages of cirrhosis, where immune tolerance and hyperresponsiveness can coexist [99]. In these patients, the balance between immune activation and tolerance is often disrupted, potentially leading to unpredictable responses to vaccination [99].

#### Challenges Posed by Cirrhosis-Associated Immune Dysfunction (CAID)

Cirrhosis-associated immune dysfunction (CAID) significantly affects vaccine immunogenicity in CLD patients [105]. CAID involves impaired T cell functionality, B cell abnormalities, and reduced complement activity, all of which can limit antibody production [106].

T cell dysfunction in CAID includes reduced proliferative capacity, impaired cytokine production, and increased expression of inhibitory receptors [107]. These defects compromise the ability of T cells to effectively support B cell activation and antibody production. B cell abnormalities in CAID involve reduced numbers of naïve B cells, impaired B cell maturation, and the increased production of autoantibodies [108]. These abnormalities can impair the ability of B cells to generate protective antibodies in response to vaccination. Reduced complement activity in CAID further compromises immune responses [109]. The complement system plays a crucial role in enhancing the antibody-mediated neutralization of pathogens and promoting inflammation. Impaired complement activity can reduce the effectiveness of vaccination [99].

Understanding CAID is essential when designing vaccination strategies for CLD patients [110]. Strategies to overcome CAID may involve using adjuvants to enhance immune responses, optimizing vaccine dosing and timing, or employing novel vaccine delivery systems.

### 4.3. Long-Term Vaccine Efficacy and Immunomonitoring

Long-term protection against HEV in CLD patients depends on the durability of protective antibody titers and the establishment of effective immune memory [54]. However, underlying immune dysfunction in CLD may compromise both of these aspects [99].

#### 4.3.1. Duration of Protective Antibody Levels and Memory Cell Formation

Assessing the longevity of anti-HEV IgG antibodies in CLD populations is essential for determining the duration of vaccine-induced protection [23]. Studies have shown that antibody titers may wane more rapidly in CLD patients compared to healthy individuals, potentially due to impaired immune responses [99].

Investigating whether sufficient memory B cell formation occurs despite underlying immune dysfunction is critical [99]. Memory B cells are essential for long-term protection, as they can rapidly differentiate into antibody-secreting cells upon re-exposure to the virus [99]. However, CAID may compromise memory B cell formation, leading to reduced long-term protection [99].

Factors influencing waning immunity include age, disease stage, and concurrent medications [99]. Those with more advanced liver disease may have weaker immune responses and faster antibody decline [99]. Immunosuppressive medications can also interfere with vaccine responses and reduce antibody persistence [99].

#### 4.3.2. Consideration for Booster Doses and Enhanced Monitoring Protocols

To enhance protection in CLD patients, strategies for booster vaccinations tailored to their specific needs should be considered [23]. Periodic revaccination schedules may be necessary to maintain adequate antibody levels and prevent HEV infection [23].

Enhanced immunomonitoring protocols are crucial for tracking both humoral and cellular immune responses over time [23]. Specific assays that could be employed include ELISA for antibody quantification and flow cytometry for memory cell phenotyping [23]. ELISA can measure the levels of anti-HEV IgG antibodies, providing an indication of humoral immunity [23]. Flow cytometry can identify and quantify memory B cells and T cells, providing insights into cellular immunity and the potential for long-term protection [23]. By implementing these strategies, healthcare professionals can optimize HEV vaccination in CLD patients and improve their long-term outcomes [111].

## 5. The Elderly

### 5.1. Impact of Immunosenescence on Vaccine Response

#### Altered T Cell Subsets and Reduced Humoral Immunity

Immunosenescence, the age-related decline in immune function, significantly impacts vaccine responses in the elderly. This decline affects both T cell and B cell compartments, leading to impaired immunity [2]. T cell function is altered, with an increase in exhausted T cells expressing programmed cell death protein 1 (PD-1+) and a reduction in CD28 co-stimulation [112,113]. The accumulation of PD-1+ T cells indicates a state of T cell exhaustion, where these cells exhibit reduced proliferation, cytokine production, and cytotoxic activity [2]. The loss of CD28 co-stimulation further impairs T cell activation and proliferation, diminishing their ability to mount effective responses to new antigens, such as those present in vaccines [112,114].

Humoral immunity also declines with age, characterized by reduced B cell diversity and lower antibody affinity maturation [2,115]. The B cell repertoire becomes less diverse, limiting the ability to produce antibodies against a wide range of antigens [116]. Furthermore, the affinity maturation process, which refines the specificity and binding strength of antibodies, is less efficient in the elderly, resulting in antibodies with lower potency [112]. These changes collectively contribute to impaired vaccine responses, as evidenced by lower seroconversion rates and shorter antibody persistence [2,117].

Studies have demonstrated impaired vaccine responses in the elderly across various vaccines [118]. For instance, influenza vaccines often exhibit reduced efficacy in older adults compared to younger individuals, with lower seroconversion rates and a decreased duration of protection [112,119]. Similarly, responses to hepatitis B vaccines are often suboptimal in the elderly, necessitating higher doses or additional booster shots to achieve adequate protection [120]. These findings highlight the challenges of achieving robust and durable immunity in older populations due to immunosenescence [121]. The implications for HEV vaccination efficacy in this population are significant, suggesting that standard vaccination protocols may not elicit optimal responses and that tailored strategies are needed to enhance immunogenicity and protection [112].

### 5.2. Strategies to Enhance Immunogenicity in the Elderly

#### 5.2.1. Use of Adjuvants (e.g., MF59, AS04) and Improved Delivery Systems

Given the impact of immunosenescence on vaccine responses in the elderly, strategies to enhance immunogenicity are crucial [122]. Adjuvants and improved delivery systems are key components in boosting immune responses in this population [2,123].

Adjuvants are substances added to vaccines to enhance the immune response to the vaccine antigen [124]. MF59 and AS04 are two well-established adjuvants that have shown promise in improving vaccine immunogenicity in older adults [112,125]. MF59 is an oil-in-water emulsion that enhances antigen presentation and stimulates both humoral and cell-mediated immunity [124,126]. It has been used in influenza vaccines for the elderly and has demonstrated improved antibody responses and clinical protection [2,127]. AS04 is a combination of aluminum salt and monophosphoryl lipid A (MPL), a TLR4 agonist that activates dendritic cells and promotes T cell responses [125]. AS04 has been used in hepatitis B and HPV vaccines and has shown enhanced immunogenicity and long-term protection in older adults [112,125].

Novel delivery systems, such as liposomes and nanoparticles, offer another avenue for improving antigen presentation and enhancing immune responses [128]. Liposomes are spherical vesicles composed of lipid bilayers that can encapsulate and deliver antigens to immune cells [129]. They can improve antigen stability, enhance cellular uptake, and promote sustained release, leading to more effective immune stimulation [130]. Nanoparticles, typically ranging in size from 1 to 100 nm, can also encapsulate antigens and deliver them to immune cells [131]. They can be designed to target specific immune cells, such as dendritic cells, and to stimulate specific immune pathways, leading to tailored immune responses [112,132].

Preclinical and clinical studies testing adjuvanted HEV vaccines in elderly populations are limited, highlighting a critical research gap [122]. However, studies with other vaccines suggest that incorporating adjuvants like MF59 or AS04 into HEV vaccines could significantly enhance immunogenicity and protection in older adults [133]. Furthermore, exploring novel delivery systems such as liposomes or nanoparticles could offer additional benefits in terms of antigen presentation and immune stimulation [134]. Further research is needed to evaluate the safety and efficacy of these strategies in the context of HEV vaccination in the elderly [135].

#### 5.2.2. Tailored Vaccination Regimens and Potential Adjustments in Dosing

Tailoring vaccination regimens and adjusting dosing strategies may be necessary to compensate for immunosenescence and improve vaccine responses in the elderly [2,117]. Higher antigen doses or additional booster doses could potentially overcome the reduced immune responsiveness in older adults [112,136].

Higher antigen doses can provide a greater stimulus to the immune system, increasing the likelihood of activating a sufficient number of immune cells to generate a protective response [137]. Studies with influenza vaccines have shown that high-dose formulations can elicit higher antibody titers and improved clinical protection in the elderly compared to standard-dose vaccines [138]. Additional booster doses can also help to boost antibody titers and prolong the duration of protection [139]. Repeated exposure to the vaccine antigen can stimulate memory B cells and T cells, leading to a more robust and durable immune response [140].

Comparing standard versus accelerated vaccination schedules in elderly cohorts may also be beneficial [141]. Accelerated schedules, involving shorter intervals between doses, can potentially lead to more rapid seroconversion and earlier protection [117]. However, they may also result in reduced long-term antibody persistence [142]. The optimal vaccination schedule should balance the need for rapid protection with the desire for durable immunity [141].

Prime-boost strategies, involving heterologous vaccination with different vaccine platforms, could also enhance immunogenicity in the elderly [120,143]. For example, priming with a DNA vaccine followed by boosting with a protein subunit vaccine can elicit strong cellular and humoral immune responses [144]. Heterologous prime-boost strategies can stimulate a broader range of immune cells and pathways, leading to more effective and durable immunity [112,145]. Clinical trials are needed to evaluate the safety and efficacy of tailored vaccination regimens, adjusted dosing strategies, and prime-boost approaches for HEV vaccination in the elderly [120,144].

### 5.3. Assessment of Cross-Genotype Protective Efficacy

Gaps in knowledge regarding elderly-specific cross-protection need to be addressed [117]. Immunosenescence may affect the ability of the elderly to mount effective cross-neutralization responses [141]. It is possible that the breadth and potency of cross-neutralizing antibodies induced by HEV vaccines are reduced in older adults compared to younger individuals [141]. This could leave the elderly more vulnerable to infection with HEV genotypes against which the vaccine provides suboptimal protection [112,146]. Further research is needed to evaluate the cross-neutralization responses induced by HEV vaccines in elderly populations and to determine whether additional strategies, such as booster doses or adjuvants, are needed to enhance cross-protection [147,148].

Table 1 summarizes the risks and potential consequences of HEV infection in pregnant women, immunocompromised individuals, CLD, and the elderly.

## 6. Current Research Gaps and Challenges

### 6.1. Inadequacy of Clinical Data for Special Populations

#### 6.1.1. Scarcity of Phase III Trial Data in Pregnant Women, Immunocompromised, and CLD Patients

HEV infection poses a significant global health challenge, especially for specific populations at risk of severe outcomes [7,23]. Despite the availability of a licensed HEV 239 vaccine in China, a critical gap exists in the availability of robust clinical data, particularly from Phase III trials, that specifically address the safety and efficacy of HEV vaccination in these vulnerable populations [6,7,18].

The scarcity of Phase III trial data in these special populations underscores the urgent need for further research to inform evidence-based HEV vaccination strategies and improve clinical outcomes. Without such data, healthcare providers face challenges in making informed decisions about HEV vaccination in these vulnerable groups, potentially leading to suboptimal prevention strategies and increased disease burden [7].

#### 6.1.2. Limited Long-Term Efficacy and Durability Studies

Beyond the scarcity of Phase III trial data in special populations, there is a significant lack of studies evaluating the long-term efficacy and durability of the protection conferred by the HEV vaccine [17]. While initial clinical trials have demonstrated the vaccine’s ability to prevent symptomatic HEV infection, the duration of protective immunity and the need for booster doses remain unclear [7,18]. Understanding the long-term effectiveness of HEV vaccines is crucial for developing optimal vaccination strategies and determining the appropriate intervals for booster immunizations [23].

The limited data on long-term efficacy are particularly relevant for special populations, who may have altered immune responses and varying rates of waning immunity [7]. Long-term studies are needed to assess the persistence of HEV-specific antibodies and memory B cells in vaccinated individuals. These studies should also evaluate the vaccine’s effectiveness against different HEV genotypes and the potential for cross-protection. Furthermore, it is important to monitor vaccinated individuals for asymptomatic HEV infections, as these can still contribute to liver damage and disease transmission. The absence of long-term efficacy data also hinders the development of cost-effective vaccination programs [7]. Without knowing how long protection lasts, it is difficult to determine the optimal age for vaccination, the need for booster doses, and the overall cost-effectiveness of HEV vaccination in different populations. Economic evaluations and cost-effectiveness studies are needed to inform policy decisions and ensure that HEV vaccines are accessible to those who need them most.

Addressing the gaps in long-term efficacy and durability data requires well-designed prospective studies with long follow-up periods. These studies should include diverse populations, including special risk groups, and should utilize standardized assays to measure antibody titers and assess vaccine effectiveness against different HEV genotypes. Collaboration between researchers, public health agencies, and vaccine manufacturers is essential to generate the data needed to optimize HEV vaccination strategies and improve public health outcomes [7,23].

### 6.2. Genotypic Variability and Cross-Protection Concerns

#### 6.2.1. Differences Between HEV Genotypes in Vaccine Performance

Cross-genotype protection is critical for HEV vaccines, given the diversity of HEV strains circulating globally [2,149]. HEV is classified into several genotypes (1, 2, 3, 4, etc.) with varying geographical distributions and transmission routes [150]. A vaccine that provides protection against only one genotype may not be sufficient to prevent HEV infection in individuals exposed to different genotypes. Therefore, it is essential to evaluate the cross-neutralization responses induced by HEV vaccines [112].

Cross-neutralization refers to the ability of antibodies elicited by a vaccine to neutralize different HEV genotypes [2,146]. This can be assessed through in vitro neutralization assays, where serum or plasma from vaccinated individuals is tested for its ability to inhibit the infectivity of different HEV genotypes in cell culture [151]. A vaccine that induces high levels of cross-neutralizing antibodies is more likely to provide broad protection against HEV infection, regardless of the infecting genotype [112,146].

Existing data on HEV vaccine cross-neutralization are limited. HEV 239, the HEV vaccine currently licensed in China, has demonstrated efficacy against genotype 1 in clinical trials [152]. However, data on its efficacy against other genotypes, particularly genotype 3, which is prevalent in industrialized countries, are scarce. Some studies suggest that HEV 239 may induce cross-neutralizing antibodies against genotype 3, but the levels of these antibodies are lower than those against genotype 1 [2,146].

The licensed HEV 239 vaccine is based on a genotype 1 strain [7]. While this vaccine has demonstrated high efficacy against genotype 1 HEV infection, its effectiveness against other genotypes, particularly genotypes 3 and 4, is not well established [21,153]. This raises concerns about the potential for reduced vaccine efficacy in regions where genotypes 3 and 4 are prevalent [21,153].

Studies have shown that antibodies induced by the genotype 1-based vaccine may exhibit reduced neutralization activity against genotypes 3 and 4 [7,154]. This suggests that the vaccine may not provide optimal protection against these genotypes. Further research is needed to assess the cross-protective efficacy of the HEV vaccine against different genotypes and to determine whether alternative vaccine formulations are needed to provide broader protection [7].

#### 6.2.2. Designing In Vitro Assays to Assess Cross-Neutralization

Assessing the cross-neutralization capacity of HEV vaccines against different genotypes requires the development and validation of reliable in vitro assays [154]. Traditional neutralization assays, which measure the ability of antibodies to inhibit viral infection of cells in vitro, can be challenging to perform with HEV due to the lack of efficient cell culture systems for all genotypes [155].

Alternative in vitro assays, such as pseudoparticle neutralization assays and reporter virus assays, have been developed to overcome these limitations [156]. These assays utilize recombinant viruses or pseudoparticles expressing HEV capsid proteins to assess the ability of antibodies to block viral entry into cells. However, these assays need to be standardized and validated to ensure their accuracy and reliability.

The design of in vitro assays for cross-neutralization should take into account the genetic diversity of HEV and the potential for antigenic variation among different genotypes. Assays should include a panel of HEV strains representing different genotypes and subtypes to assess the breadth of neutralizing antibody responses [157]. It is also important to consider the potential for the antibody-dependent enhancement (ADE) of HEV infection, in which antibodies may enhance viral entry into cells rather than neutralizing it.

In addition to in vitro assays, in vivo studies in animal models are needed to assess the cross-protective efficacy of HEV vaccines against different genotypes [158]. Animal models, such as non-human primates and mice, can be used to evaluate the ability of vaccines to protect against HEV infection and liver disease. However, it is important to consider the limitations of animal models and to interpret the results cautiously.

### 6.3. WHO Prequalification and Global Accessibility Issues

The WHO prequalification process plays a crucial role in ensuring the quality, safety, and efficacy of vaccines and medicines used in international immunization programs. WHO prequalification involves a rigorous assessment of the manufacturing process, clinical trial data, and post-marketing surveillance data to ensure that the product meets international standards [7].

The licensed HEV 239 vaccine has not yet been prequalified by the WHO [159]. This limits its accessibility to many low- and middle-income countries where the disease burden is highest. Without WHO prequalification, many international organizations and national governments are reluctant to procure and distribute the vaccine [7].

The lack of WHO prequalification is due to several factors, including the limited availability of data on the vaccine’s safety and efficacy in diverse populations and the lack of a comprehensive technology transfer package to enable local production in developing countries [160]. Addressing these issues requires collaboration between the vaccine manufacturer, the WHO, and other stakeholders [7].

## 7. Future Directions in HEV Vaccination Research

### 7.1. Expanding Clinical Trial Networks and Special Population Coverage

#### 7.1.1. Designing Multi-Center Trials for High-Risk Groups

Multi-center clinical trials are crucial for gathering robust and diverse data on the safety and efficacy of HEV vaccines across different demographics [7]. High-risk groups, such as pregnant women, immunocompromised patients, and those with chronic liver disease, require specific attention due to their increased vulnerability to severe HEV infection outcomes [7,23]. By involving multiple institutions and research centers, these trials can enhance the generalizability of findings and account for regional variations in HEV genotypes and transmission patterns [23]. Standardized protocols are essential to ensure consistency and comparability of data across different sites [41]. These protocols should include uniform diagnostic criteria, standardized methods for assessing vaccine immunogenicity and safety, and clear guidelines for managing adverse events [41]. Collaborative efforts among institutions can also facilitate the sharing of expertise, resources, and data, thereby accelerating the pace of HEV vaccine research and development [23]. Multi-center trials allow for the inclusion of a more representative sample of the target population, capturing the heterogeneity in genetic backgrounds, co-morbidities, and environmental exposures that may influence vaccine responses [7]. Furthermore, these trials can provide valuable insights into the cost-effectiveness of HEV vaccination strategies in different healthcare settings, informing policy decisions and resource allocation [6,54].

#### 7.1.2. Optimizing Timing Strategies (e.g., Vaccination Prior to Exposure)

The strategic timing of vaccinations is particularly important for high-risk groups to maximize protection against HEV infection [54]. Prophylactic vaccination, administered before potential exposure events such as outbreaks in endemic regions, can provide timely immunity and prevent severe disease outcomes [3]. For pregnant women, vaccination prior to conception may be an optimal strategy to protect both the mother and the fetus, avoiding any potential risks associated with vaccination during pregnancy [37]. This approach requires effective screening programs to identify women of childbearing age and offer vaccination as part of pre-conception care [23]. In immunocompromised patients, vaccination should be ideally administered before the initiation of immunosuppressive therapies, allowing for a more robust immune response [161]. However, if this is not possible, adjusting the timing of vaccination to coincide with periods of reduced immunosuppression may improve vaccine immunogenicity [162]. Existing guidelines and recommendations for vaccination schedules should be tailored to specific populations, considering factors such as age, immune status, and risk of exposure [111]. For instance, in elderly individuals, a higher vaccine dose or the use of adjuvants may be necessary to overcome immunosenescence and enhance antibody responses [23]. Vaccination campaigns targeting specific communities or occupational groups at high risk of HEV exposure, such as healthcare workers and sanitation workers, can also benefit from optimized timing strategies to coincide with periods of increased risk [163].

### 7.2. Novel Vaccine Platforms and Technological Innovations

#### 7.2.1. Development of Broad-Spectrum or Multi-Epitope Vaccines

The development of vaccines targeting multiple strains or genotypes of HEV is crucial for providing broader protection against the virus [164]. HEV exhibits significant genetic diversity, with four major genotypes (1–4) and several sub-genotypes that vary in their geographical distribution and clinical manifestations [4]. Current vaccines, such as HEV 239, are based on a single genotype (genotype 1) and may not provide optimal protection against other genotypes [18,153]. Recent advances in immunology and virology have opened new avenues for creating vaccines incorporating multiple epitopes from different HEV genotypes [153]. Multi-epitope vaccines can elicit broadly neutralizing antibodies that target conserved regions of the viral capsid protein, pORF2, which are critical for viral entry and replication [153]. These vaccines can be designed using bioinformatics tools to identify and select epitopes that are recognized by a wide range of human leukocyte antigen (HLA) alleles, ensuring broad population coverage [2]. Another approach involves the development of chimeric virus-like particles (VLPs) displaying epitopes from multiple HEV genotypes [165]. VLPs are highly immunogenic and can induce strong antibody and T cell responses without the risk of viral replication [21]. Broad-spectrum vaccines could also incorporate adjuvants that enhance cross-reactive immune responses, further improving their protective efficacy against diverse HEV strains [23].

#### 7.2.2. Exploration of Advanced Delivery Systems (Liposomes, Nanoparticles, DNA Platforms)

Innovations in vaccine delivery systems can improve the stability, efficacy, and immunogenicity of HEV vaccines [166]. Traditional vaccine formulations may suffer from poor stability, limited antigen presentation, and suboptimal immune responses, particularly in vulnerable populations [2]. Nanotechnology-based delivery systems, such as liposomes and nanoparticles, offer several advantages, including enhanced antigen stability, targeted delivery to immune cells, and improved induction of cellular and humoral immunity [37]. Liposomes are spherical vesicles composed of lipid bilayers that can encapsulate and protect vaccine antigens, facilitating their delivery to antigen-presenting cells (APCs) [37]. Nanoparticles, made from biodegradable polymers or inorganic materials, can be engineered to control the size, shape, and surface properties of vaccine carriers, optimizing their interaction with the immune system [165]. DNA vaccines represent another cutting-edge approach for HEV vaccination [166]. These vaccines involve the direct introduction of plasmid DNA encoding HEV antigens into host cells, leading to in situ antigen expression and induction of both antibody and T cell responses [65]. DNA vaccines are relatively easy to manufacture, are stable at room temperature, and can elicit long-lasting immunity [65]. Advanced delivery systems can also be combined with adjuvants to further enhance vaccine immunogenicity [23]. For example, liposomes or nanoparticles can be formulated with Toll-like receptor (TLR) agonists to stimulate innate immune responses and promote adaptive immunity [2]. The use of prime-boost strategies, involving sequential immunization with different vaccine platforms, can also improve the breadth and durability of immune responses against HEV [23].

### 7.3. Integration with Public Health and Policy Strategies

#### 7.3.1. Economic Evaluations and Cost-Effectiveness Studies for Global Uptake

Economic evaluations of HEV vaccination programs are essential to assess their cost-effectiveness and inform resource allocation decisions [54]. These evaluations should consider the direct and indirect costs associated with HEV infection, including healthcare expenses, lost productivity, and impact on quality of life [6]. Cost-effectiveness analyses can compare different vaccination strategies, such as universal vaccination, the targeted vaccination of high-risk groups, and reactive vaccination during outbreaks [41]. The results of these analyses can guide policy decisions regarding the implementation of HEV vaccination programs in different socioeconomic contexts [6]. Factors such as vaccine price, delivery costs, and target population size should be taken into account [6]. Economic evaluations should also consider the potential long-term benefits of HEV vaccination, such as the reduced incidence of chronic liver disease and liver cancer [161]. Sensitivity analyses can be used to assess the impact of uncertainty in key parameters, such as vaccine efficacy and the duration of protection, on the overall cost-effectiveness [41]. The findings of economic evaluations should be communicated to policymakers and healthcare providers to promote the adoption of evidence-based vaccination strategies [23].

#### 7.3.2. Development of International Collaborations and Funding Mechanisms (e.g., GAVI Models)

International collaboration is crucial to promote HEV vaccination globally, particularly in low- and middle-income countries where the disease burden is highest [6]. This collaboration should involve the sharing of scientific knowledge, technical expertise, and financial resources [23]. Successful models like GAVI, the Vaccine Alliance, have facilitated vaccine access in low-income countries by pooling resources from donor governments, international organizations, and the private sector [23]. Similar frameworks or strategies could be developed to support HEV vaccination initiatives, particularly in vulnerable populations [6]. Funding mechanisms should be established to support the research and development of new HEV vaccines, as well as the procurement and distribution of existing vaccines [7]. International organizations, such as the World Health Organization (WHO), can play a key role in coordinating global efforts to combat HEV [6]. This includes setting standards for vaccine quality and safety, providing technical assistance to countries implementing vaccination programs, and advocating for increased investment in HEV control [6]. Public–private partnerships can also be leveraged to accelerate the development and deployment of HEV vaccines [165]. These partnerships can bring together the expertise and resources of pharmaceutical companies, research institutions, and government agencies to address the challenges of HEV prevention and control [150]. By fostering international collaboration and establishing sustainable funding mechanisms, the global community can work together to reduce the burden of HEV and improve the health and well-being of vulnerable populations [150].

Table 2 outlines the gaps in current research and potential future directions for HEV vaccination, specifically addressing the unique needs of the special populations. It provides a comprehensive overview of the unique challenges faced by these populations and the research gaps that need to be addressed to improve HEV vaccination strategies.

## 8. Conclusions

HEV continues to pose a significant health risk, particularly among vulnerable populations such as pregnant women, immunocompromised individuals, patients with CLD, and the elderly. While the HEV 239 vaccine has demonstrated efficacy in the general population, there are still notable research gaps that need to be addressed. These gaps include the lack of Phase III clinical trial data for special populations, which has left critical questions about safety, long-term efficacy, and optimal vaccination strategies unanswered. The lack of data specific to high-risk groups is a major limitation, as these populations have divergent immune responses that require specialized approaches.

Additionally, long-term efficacy studies are needed to determine whether the protection offered by the HEV239 vaccine persists over time, especially for these vulnerable groups. The accessibility of the vaccine is also limited, particularly in low-resource settings, where regulatory, economic, and logistical challenges must be overcome. Despite these challenges, HEV vaccination holds considerable promise for reducing the disease burden in high-risk populations, but ongoing monitoring and tailored strategies are essential for maximizing its effectiveness.

Several limitations of this review should be considered. First, there is an inherent limitation in potentially omitting unpublished studies or those published in non-English languages. Second, granular cost-effectiveness data have been excluded due to the lack of comprehensive sources. Moreover, challenges exist in extrapolating findings from general populations to special populations, where clinical trials are often limited or absent. Finally, the insights specific to vulnerable populations should be interpreted with caution, particularly when data are derived indirectly or from animal models.

In conclusion, while HEV vaccination offers significant promise, it is clear that further research is needed to address the gaps in safety and efficacy for special populations. Efforts to improve vaccine accessibility, optimize vaccine strategies for high-risk groups, and overcome barriers to global distribution will be essential in reducing the global burden of HEV.

## Figures and Tables

**Table 1:** Risk and Impact of HEV Infection in Special Populations.

Population	Increased Risk Factors	Potential Consequences	Vaccine Efficacy Concerns
Pregnant Women	Immune modulation (Th2 bias), high viral loads	Fulminant hepatitis, maternal and fetal mortality, obstetric complications	Insufficient clinical data, need for vaccine safety trials
Immunocompromised Individuals	Immunosuppressive therapies (e.g., tacrolimus, MMF), poor immune response	Chronic infection, liver fibrosis, cirrhosis	Reduced vaccine response, need for tailored strategies
Patients with Chronic Liver Disease (CLD)	Pre-existing liver damage, immune dysfunction	Accelerated liver decompensation, acute-on-chronic liver failure	Vaccine response may be impaired, ongoing immune dysfunction
Elderly	Immunosenescence, altered immune response	Reduced vaccine efficacy, shorter protection duration	Need for higher doses or adjuvants, concerns about cross-protection

**Table 2:** Current Research Gaps and Future Directions in HEV Vaccination Research.

Research Gap	Current Challenges	Future Directions
Clinical Data in Special Populations	Limited Phase III trial data in pregnant women, immunocompromised individuals, and CLD patients	Multi-center trials in high-risk groups, including long-term safety and efficacy studies
Vaccine Response in Immunocompromised Groups	Poor vaccine efficacy due to immunosuppressive therapy	Sequential “vaccine-immunomodulator” strategies, personalized immunization schedules
Vaccine Efficacy in Elderly Populations	Reduced immune response due to immunosenescence	Use of adjuvants (e.g., MF59, AS04), exploration of higher vaccine doses or booster shots
Cross-Genotype Protection	Limited cross-neutralization of HEV genotypes	Development of broad-spectrum vaccines, incorporation of multiple genotypes and epitopes
Global Accessibility and Prequalification	Limited access in low-income countries due to lack of WHO prequalification	Global collaborations for vaccine distribution, funding mechanisms for vaccine accessibility

## Data Availability

Data sharing is not applicable to this article.
